# Magnetic Resonance Imaging Texture Analysis Based on Intraosseous and Extraosseous Lesions to Predict Prognosis in Patients with Osteosarcoma

**DOI:** 10.3390/diagnostics14222562

**Published:** 2024-11-15

**Authors:** Yu Mori, Hainan Ren, Naoko Mori, Munenori Watanuki, Shin Hitachi, Mika Watanabe, Shunji Mugikura, Kei Takase

**Affiliations:** 1 Department of Orthopaedic Surgery, School of Medicine, Tohoku University Graduate, Sendai 980-8574, Japan; yu.mori.c4@tohoku.ac.jp (Y.M.); mwata@ortho.med.tohoku.ac.jp (M.W.); 2 Department of Diagnostic Radiology, School of Medicine, Tohoku University Graduate, Sendai 980-8574, Japan; merylren1994@163.com (H.R.); shin.hitachi.d4@tohoku.ac.jp (S.H.); mugi844@gmail.com (S.M.); ktakase@rad.med.tohoku.ac.jp (K.T.); 3 Department of Radiology, School of Medicine, Akita University Graduate, Akita 010-8543, Japan; 4 Department of Pathology, School of Medicine, Tohoku University Graduate, Sendai 980-8574, Japan; mkawatan@patholo2.med.tohoku.ac.jp; 5 Division of Image Statistics, Tohoku Medical Megabank Organization, Tohoku University, Sendai 980-8574, Japan

**Keywords:** osteosarcoma, magnetic resonance imaging, texture analysis, histological pattern, survival

## Abstract

**Objectives:** To construct an optimal magnetic resonance imaging (MRI) texture model to evaluate histological patterns and predict prognosis in patients with osteosarcoma (OS). **Methods:** Thirty-four patients underwent pretreatment MRI and were diagnosed as having OS by surgical resection or biopsy between September 2008 and June 2018. Histological patterns and 3-year survival were recorded. Manual segmentation was performed in intraosseous, extraosseous, and entire lesions on T1-weighted, T2-weighted, and contrast-enhanced T1-weighted images to extract texture features and perform principal component analysis. A support vector machine algorithm with 3-fold cross-validation was used to construct and validate the models. The area under the receiver operating characteristic curve (AUC) was calculated to evaluate diagnostic performance in evaluating histological patterns and 3-year survival. **Results:** Eight patients were chondroblastic and the remaining twenty-six patients were non-chondroblastic patterns. Twenty-seven patients were 3-year survivors, and the remaining seven patients were non-survivors. In discriminating chondroblastic from non-chondroblastic patterns, the model from extraosseous lesions on the T2-weighted images showed the highest diagnostic performance (AUCs of 0.94 and 0.89 in the training and validation sets). The model from intraosseous lesions on the T1-weighted images showed the highest diagnostic performance in discriminating 3-year non-survivors from survivors (AUCs of 0.99 and 0.88 in the training and validation sets) with a sensitivity, specificity, positive predictive value, and negative predictive value of 85.7%, 92.6%, 75.0%, and 96.2%, respectively. **Conclusions:** The texture models of extraosseous lesions on T2-weighted images can discriminate the chondroblastic pattern from non-chondroblastic patterns, while the texture models of intraosseous lesions on T1-weighted images can discriminate 3-year non-survivors from survivors.

## 1. Introduction

Osteosarcoma (OS) is the most frequent primary malignant bone tumour, with its highest prevalence in children and adolescents and the second highest prevalence in adults aged 70–80 years [1,2,3]. Although OS accounts for only about 5% of childhood and adolescent cancers, it contributes significantly to the mortality rate from childhood cancer, especially since the 1970s, when the survival rate did not improve compared to other childhood cancers [4,5].

The advent of chemotherapy in the second half of the 20th century enabled the introduction of neoadjuvant and adjuvant chemotherapy during the 1970s and 1980s. This led to improved overall survival rates in osteosarcoma patients with nonmetastatic disease at diagnosis, reaching 70% [6,7]. However, it did not significantly improve outcomes for patients presenting with macrometastases at diagnosis or those with relapsed disease, as both groups continue to exhibit survival rates of only 20% [7]. Unlike many other pediatric cancers [5], empirical treatment intensification for osteosarcoma has failed to substantially improve survival rates over the past four decades. Thus, it seems paramount to unravel the underlying mechanisms that determine chemotherapy susceptibility and resistance in osteosarcoma. There is hope for the development of a new prognostic prediction method that combines histological and imaging examinations to predict the efficacy of chemotherapy for osteosarcoma.

Histologically, OS exhibits a mixed histological pattern, including osteoblastic, chondroblastic, and fibroblastic components [8,9,10,11]. Several studies have reported that patients with chondroblastic patterns (chondroblast-dominant differentiation) may be less responsive to chemotherapy and have a worse prognosis than patients with non-chondroblastic patterns [2,12,13,14].

On the T2-weighted (T2W) imaging of magnetic resonance imaging (MRI), the chondroblastic pattern visually exhibits high signal intensity compared to non-chondrobalstic patterns (osteoblastic- and fibroblastic component-dominant differentiation) [15]. However, the chondroblastic pattern could not be discriminated from non-chondroblastic patterns by visual imaging findings due to the mixed histological nature of OS [9,16,17,18,19]. Furthermore, to our knowledge, no study has reported that visual imaging findings can predict prognosis.

OS often occurs in long bones and presents as intraosseous and extraosseous lesions [11,16,20]. MRI has proven sensitive to evaluate the extent of the entire OS lesion, including intraosseous and extraosseous lesions [16]. Holscher et al. have analysed the volume and signal intensity of the intra- and extraosseous lesions by placing a region of interest on MRI and showed that the signal intensity of extraosseous lesions correlated with response to chemotherapy in patients with OS [21,22].

Texture analysis, an effective tool that quantitatively evaluates lesion heterogeneity by measuring spatial variations in the signal intensity, has been extensively used in other areas such as soft tissue sarcoma, breast cancer, and placenta accrete spectrum [23,24,25,26]. In the field of orthopaedics, there are reports on the diagnosis of osteonecrosis of the mandible, the diagnosis of metastatic bone tumours, and the differentiation between giant cell tumours and aneurysmal bone cysts using texture analysis [27,28,29]. The grey-level co-occurrence matrix method, one of the texture analyses proposed by Haralick et al., evaluates the arrangement and interrelation among grey-level pixel intensities [30]. In OS, previous studies have reported the utility of texture analysis to predict the risk of metastases, response to chemotherapy, and prognosis [23,31,32]. In those studies, segmentations were performed on the entire OS lesion to extract texture features. To our knowledge, no study has identified the usefulness of texture features extracted from intraosseous, extraosseous, and entire lesion compartments on MRI in evaluating histological patterns and predicting prognosis.

This study aimed to evaluate the relationship between MRI-based texture analysis and histological patterns and analyse the diagnostic performance of MRI-based texture analysis to predict 3-year survival in patients with OS.

## 2. Materials and Methods

### 2.1. Patients

This study is a proof-of-concept study for the construction of a texture model analysis for long bone OS. The institutional review board approved this retrospective study and waived the requirement of informed consent. Eighty-two consecutive patients underwent pretreatment MRI and were diagnosed with primary OS by surgical resection or biopsy between September 2008 and June 2018. All the patients underwent CT for >3 years every 6 months. Of these, 48 patients were excluded for the following reasons: (a) flat bone OS, such as facial bone (*n* = 13), ribs (*n* = 8), shoulder or scapular (*n* = 14), pelvis (*n* = 4), and toe (*n* = 3) due to different responses to chemotherapy and different prognoses of flat bone OS from long bone OS [33,34]; (b) patients with other histological OS subtypes, such as parosteal OS (*n* = 1), telangiectatic OS (*n* = 1), small-cell OS (*n* = 2), and giant-cell rich OS (*n* = 2). OS occurring in flat bones and other histological types of OS were excluded to maintain the accuracy of the prognostic prediction model, as they often differ in pathology and treatment response compared to long bone OS [33,34]. This approach also ensures that the analysis is based on a more homogeneous patient group, thereby enhancing the reliability of the predictive model. A total of 34 patients with conventional long bone OS were enrolled in this study. The 3-year survival was designated as the endpoint in this study. The patients were divided into non-survivor and survivor groups. During a 3-year follow-up, lung metastases were diagnosed based on the appearance of typical pulmonary findings on CT or proved by biopsy. Twenty-one patients were diagnosed with lung metastases, and the remaining 13 patients were free from lung metastases during the 3-year follow-up.

Patient characteristics, including sex, age, tumour location, tumour length, lung metastasis, the time of detection of lung metastasis, surgical resection, histological patterns (chondroblastic and non-chondroblastic), chemotherapy, American Joint Committee on Cancer (AJCC) staging at presentation, and the time interval between pretreatment MRI and surgical resection, were recorded [35,36]. The tumour length was defined as the longest diameter of the OS lesion measured in the axial, sagittal, and coronal planes on the T1W image (a cut-off length of 80 mm was used according to the AJCC staging) [37].

### 2.2. Image Acquisition

The MRI examinations of 22 patients were performed using a 3.0 T system (MAGNETOM Trio, A Tim System, Siemens Healthcare GmbH, Erlangen, Germany) with our standard protocol for evaluating the extent of OS lesions. The patients were imaged in the feet-first supine position with 12 coil elements. The standard MRI protocol was composed of a two-dimensional (2D) axial turbo spin echo (TSE) T1W sequence (repetition time (TR)/echo time (TE), 600/7 ms; FOV, 100 mm; slice thickness, 5 mm; matrix size, 408 × 408; bandwidth = 200 Hz; flip angle = 90°; number of slices, 20; temporal resolution, 238 s; fat suppression method, chemical shift selective [CHESS] or short tau inversion recovery [STIR]) and axial TSE T2W sequence (TR/TE, 4000/75 ms; FOV, 100 mm; slice thickness, 5 mm; matrix size, 512 × 512; bandwidth, 200 Hz; flip angle, 150°; fat suppression method, CHESS or STIR). Contrast-enhanced MRI was performed using a 2D axial TSE T1W sequence (TR/TE, 782/10 ms; FOV, 100 mm; slice thickness, 5 mm; matrix size, 408 × 408; bandwidth, 200 Hz; flip angle, 150°; fat suppression method, SPGR) with the manual injection of meglumine gadoterate (Magnescope, Fuji Pharma Co. Ltd., Tokyo, Japan) administered intravenously at a dose of 0.1 mmol/kg body weight, followed by a 20 mL saline flush at a rate of 2 mL/s.

The remaining MRI examinations of 12 patients were performed on a 1.5 T system (Achieva; Philips Healthcare, the Netherlands). The patients were imaged with their feet in the first supine position with a SENSE body coil. The standard MRI protocol was composed of a 2D axial spin echo (SE) T1W sequence (TR/TE, 492/13 ms; FOV, 100 mm; slice thickness, 5 mm; matrix size, 304 × 304; bandwidth, 287 Hz; flip angle, 90°; number of slices, 20; temporal resolution, 238 s; fat suppression method, CHESS or STIR) and the axial SE T2W sequence (TR/TE, 4545/100 ms; FOV, 96 mm; slice thickness, 5 mm; matrix size, 336 × 336; bandwidth, 150 Hz; flip angle, 150°; fat suppression method, CHESS or STIR). Contrast-enhanced MRI was performed using a 2D axial SE T1W sequence (TR/TE, 600/10 ms; FOV, 96 mm; slice thickness, 5 mm; matrix size, 288 × 288; bandwidth, 243 Hz; flip angle, 90°; fat suppression method, SPGR) with the manual injection of meglumine gadoterate (Magnescope, Fuji Pharma Co. Ltd., Tokyo, Japan) administered intravenously at a dose of 0.1 mmol/kg body weight, followed by a 20 mL saline flush at a rate of 2 mL/s.

### 2.3. Image Analysis

One experienced orthopaedist (with 15 years of experience in musculoskeletal diagnosis and treatment) and one experienced radiologist (with 17 years of experience in radiology) who were blinded to the clinical information of the patients, other imaging findings (i.e., X-ray and bone CT), and histological diagnoses performed the manual segmentation.

#### 2.3.1. Tumour Segmentation

The MRI DICOM data of 34 patients were transferred from a commercially available medical workstation (HMC Viewer Ver. V1.0.0, Hitachi, Tokyo, Japan) using a personal computer. Since DICOM data contains identifiable metadata, we anonymised the DICOM files prior to the data transfer to protect patient privacy in this study. Specifically, identifiable information such as patient name, ID number, date of birth, and examination date were completely removed. This process ensured that the DICOM files were accessible to reviewers and contained no personal information, thus safeguarding privacy. Tumour segmentation was performed manually by placing the 2D regions of interest (ROIs) slice-by-slice using LIFEx Ver.6.30 [38]. In this study, we did not perform image registration (image alignment) in order to minimise the impact of positional misalignment between each MRI sequence, as we prioritised ensuring the accuracy of manual segmentation.

**Figure 1:**
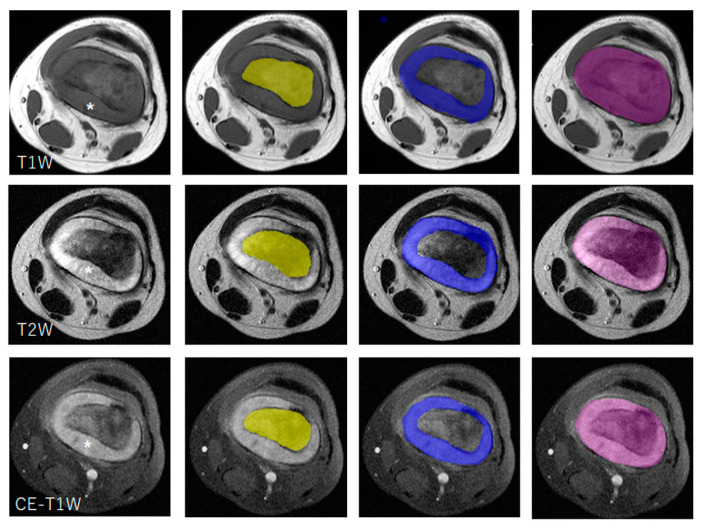
A 14-year-old female with histologically proven osteosarcoma (chondroblastic pattern) in the left femur. Manual segmentations for different lesion compartments are performed separately on the T1W, T2W, and CE-T1W images. For intraosseous lesions, the regions of interest (ROIs) were placed on the T1W images with reference to the T2W and CE-T1W images (yellow). Intramedullary heterogeneous hypo-intensity to iso-intensity on the T1W images and patchy hypo-intensity to iso-intensity on the T2W images are considered intraosseous lesions. For extraosseous lesions, the ROIs are placed on the T2W images with reference to the T1W and CE-T1W images (blue). Soft tissue masses showing iso-intensity on the T1W images and iso-intensity to hyper-intensity on the T2W images with enhancement on the CE-T1W images are considered extraosseous lesions (asterisk). The involved cortical bone showing iso-to-hyper-intensity alongside the intramedullary cavity on the T1W and T2W images is determined as extraosseous lesions. Finally, the ROIs for intraosseous and extraosseous lesions are combined to obtain the ROI for the entire lesion (purple).

For intraosseous lesions, the ROIs were placed on the T1W images with reference to the T2W and CE-T1W images (Figure 1). The ROIs were copied and applied to the T2W and CE-T1W images. Intramedullary heterogeneous hypo-intensity to iso-intensity on the T1W images and patchy hypo-intensity to iso-intensity on the T2W images were considered intraosseous lesions. For extraosseous lesions, the ROIs were placed on the T2W images with reference to the T1W and CE-T1W images (Figure 1). The ROIs were copied and applied to the T1W and CE-T1W images. The soft tissue masses that show iso-intensity on the T1W images and iso-intensity to hyper-intensity on the T2W images with enhancement on the CE-T1W images were considered extraosseous lesions. The ROIs for intraosseous and extraosseous lesions were then combined to obtain the ROIs for entire lesions (Figure 1). The ROIs of entire lesions were applied to the T1W, T2W, and CE-T1W images. According to previous studies, cortical bone (identified as hypo-intensity along with hyperintense marrow on T1W and T2W images) did not belong to intramedullary components [23,39,40,41,42]. Therefore, the cortical bone was included in the ROIs of the extraosseous lesions in our study.

#### 2.3.2. Texture Feature Processing

**Figure 2:**
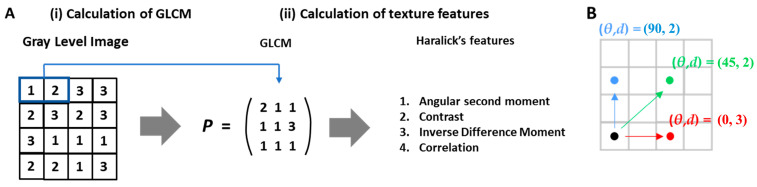
Haralick’s texture features extraction conceptual diagram. (**A**) Conceptual representation of texture features extracted from the grey-level co-occurrence matrix (GLCM), illustrating the frequency calculation of relative grey-level values between pixels in various directions and distances. (**B**) Explanation of derived texture indicators such as contrast, uniformity, and entropy based on the GLCM data.

A conceptual diagram of Haralick’s texture feature extraction is presented to explain MRI texture analysis [30] (Figure 2). Texture features were extracted separately from the ROIs of the intraosseous, extraosseous, and entire lesions on the T1W, T2W, and CE-T1W images. Two-dimensional processing was used to calculate the texture features: 2D pixels and their associated x- and y-directions were included. A 2D ROI should include at least 16 pixels [39]. The texture features were continuously calculated from the ROIs for all the selected slices and the averages of these texture features were used. Eighty-six texture features were extracted for each tumour segmentation (Table S1).

### 2.4. Statistical Analysis

#### 2.4.1. For Patient Characteristics

Sex, tumour location, tumour length, lung metastasis, time of detection of lung metastasis, surgical resection, histological patterns, chemotherapy, and AJCC staging at presentation were compared between the non-survivor and survivor groups using Fisher’s exact test. The age of the patients and the time interval between pretreatment MRI and surgical resection were compared between the non-survivor and survivor groups using the Wilcoxon signed-rank test.

#### 2.4.2. For Texture Analysis

##### Texture Feature Reduction and Selection

**Figure 3:**
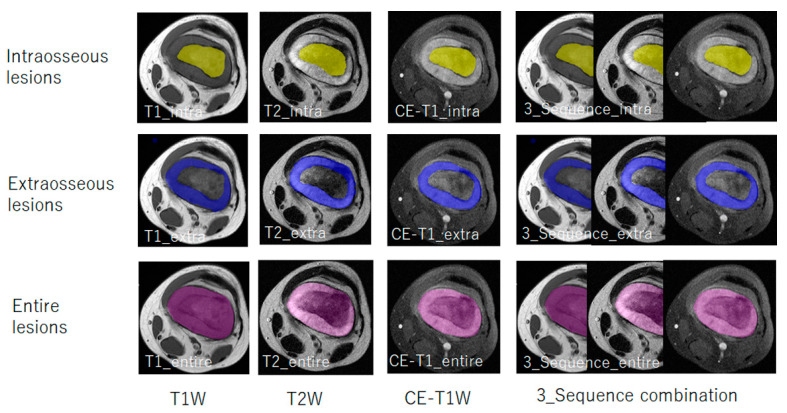
Model construction from each lesion compartment for each sequence and 3-sequence combination. A total of 12 texture models are developed using the texture features extracted from each lesion compartment in each sequence (nine models) and the texture features extracted from each lesion compartment in the 3-sequence combination (three models). Four models are constructed to evaluate intraosseous lesions—T1_intra, T2_intra, CE-T1_intra, and 3-Sequence_intra; four models to evaluate extraosseous lesions—T1_extra, T2_extra, CE-T1_extra, and 3-Sequence_extra; and four models for entire lesions—T1_entire, T2_entire, CE-T1_entire, and 3-Sequence_entire.

Since we aimed to evaluate the usefulness of the texture features extracted from the intraosseous, extraosseous, and entire lesion compartments on MRI separately to evaluate histological patterns and predict 3-year survival, we constructed texture models using the texture features extracted from each lesion compartment in each sequence (Figure 3). Additionally, we constructed texture models using the sum of the texture features extracted from each lesion compartment in the three sequences (Figure 3). A principal component analysis (PCA) was applied to fuse and reduce the texture features extracted from the ROIs to principal components (PCs). PCA is a technique that reduces a high-dimensional dataset to a low-dimensional dataset while retaining most of the variation in the data and expresses the entire dataset in terms of eigenvalues, eigenvectors, and contribution ratios. The first PC describes most of the variance in the dataset and is often considered the most important PC [43]. The eigenvalue shows the amount of information each PC represents in the total amount of information (such as variation in data) in the dataset. The first PC takes the largest eigenvalue and then gradually decreases [44]. PCA was performed until the cumulative contribution ratio of the last PC was closest to 80%, and these PCs were selected to develop the texture models.

##### Texture Model Construction and K-Fold Cross-Validation

A support vector machine (SVM) algorithm with radical basis function kernels (*K*) with the lowest minimum misclassification rate was used to develop texture models to discriminate between the chondroblastic and non-chondroblastic groups and to discriminate between the non-survivor and survivor groups with the selected PCs as input factors [45]. Equation *K* (*x*, *y*) = exp (−γ∥*x* − *y*∥^2^) was used, where *x* indicates the selected PCs significantly correlating with the discrimination performance, *y* represents the kernel centre, and *γ* is the parameter in the kernel function [46]. The *γ* parameter was set at 1 and the cost parameter (the penalty associated with misclassification and observation) at 0.1. Training and validation sets were randomly obtained using a k-fold cross-validation method. The training and validation sets were constructed by splitting the dataset into *k* = 3 equally sized folds where one is reserved for validation and the others are combined for training [25,47]. The process was repeated three times [48,49]. In this 3-fold cross-validation, we divided the data by patient, ensuring that data from different patients were assigned to either the training or validation sets. Specifically, different MRI sequences of the same patient (e.g., T1, T2, and contrast-enhanced T1) were always placed within the same set to prevent any overlap of data from the same patient between the training and validation sets. This approach minimised model bias by ensuring that the anatomical and texture information from the same patient did not cross between the training and validation stages. The diagnostic performances of these texture models in discriminating between the chondroblastic and non-chondroblastic groups and between non-survivor and survivor groups were evaluated by receiver operating characteristic (ROC) curve analysis with the area under the curve (AUC). In this study, we constructed separate models for each group (chondroblastic vs. non-chondroblastic, and non-survivors vs. survivors), training a total of 12 models. Specifically, the models were created for each lesion area (intraosseous, extraosseous, and whole lesion) and each MRI sequence (T1, T2, and contrast-enhanced T1), and these were evaluated through cross-validation. The mean AUCs were calculated in the training and validation sets. The model with the highest AUC in the validation sets was considered the optimal model. The sensitivity, specificity, positive predictive value (PPV), and negative predictive value (NPV) of the developed texture models to evaluate the 3-year survival were calculated at a cut-off point that maximised the value of the Youden index. Statistical analyses were performed using JMP Pro 16 (SAS Institute, Cary, NC, USA). A *p*-value of less than 0.05 was considered statistically significant.

## 3. Results

During the 3-year follow-up period, seven non-survivors and twenty-seven survivors were identified in this study. Eight patients were identified as chondroblastic patterns, and the remaining were identified as non-chondroblastic patterns. Among the 34 patients, 2 patients did not receive neoadjuvant chemotherapy due to advanced age (95 and 87 years). The remaining patients received preoperative chemotherapy using two protocols: neoadjuvant chemotherapy for OS in Japan (NECO-95J) (*n* = 1) and Japan Clinical Oncology Group 0905 (JCOG0905) (*n* = 31) [50,51]. The sex distribution was 10 males and 17 females in the survival group, and 0 males and 7 females in the non-survival group. The median age was 27 years in the survival group and 40 years in the non-survival group. The most common tumour site was the femur in both groups, accounting for more than 70% of the cases. In the survival group, 21 out of 27 patients had a tumour with a long diameter of over 80 mm, while 6 had a long diameter of less than 80 mm. In the non-survival group, 6 out of 7 patients had a tumour with a long diameter of over 80 mm. Lung metastasis was identified as an initial symptom in two patients in the survival group, but none in the non-survival group. Most cases of lung metastasis were identified by CT imaging or biopsy during the course of the disease. In the survival group, 9 out of 27 patients underwent amputation of the affected limb, compared to 4 out of 7 patients in the non-survival group. The predominant histological pattern in both groups was non-chondroblastic, observed in 20 out of 27 cases in the survival group and 6 out of 7 cases in the non-survival group. Significant differences in lung metastasis (*p* = 0.0051) were found between the 3-year non-survivor and survivor groups. No significant differences were found in sex, age, tumour location, tumour length, the time of detection of lung metastasis, surgical resection, histological patterns (chondroblastic and non-chondroblastic), chemotherapy, AJCC staging at presentation, and the time interval between pretreatment MRI and surgical resection (Table 1).

A total of 12 texture models were developed (Table 2). A total of four models were developed to evaluate intraosseous lesions: T1_intra, T2_intra, CE-T1_intra, and 3-Sequence_intra. Similarly, four distinct models were constructed for the assessment of extraosseous lesions: T1_extra, T2_extra, CE-T1_extra, and 3-Sequence_extra. Additionally, four comprehensive models were designed to assess all lesion types: T1_entire, T2_entire, CE-T1_entire, and 3-Sequence_entire.

**Figure 4:**
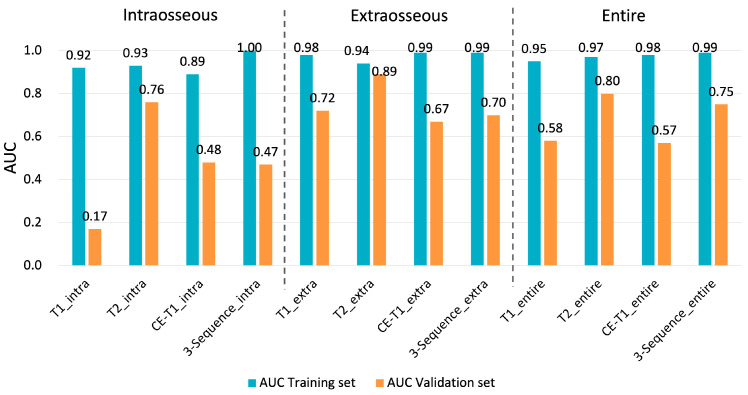
Diagnostic performance of the 12 texture models using the support vector machine (SVM) algorithm for the training and validation sets in discriminating the chondroblastic pattern from non-chondroblastic patterns (8 vs. 26). Model T1_intra is constructed using the texture features extracted from intraosseous lesions on the T1-weighted images. The other models are constructed similarly. Model T2_extra showed the highest diagnostic performance among the 12 models (AUCs of 0.94 and 0.89 in the training and validation sets).

**Figure 5:**
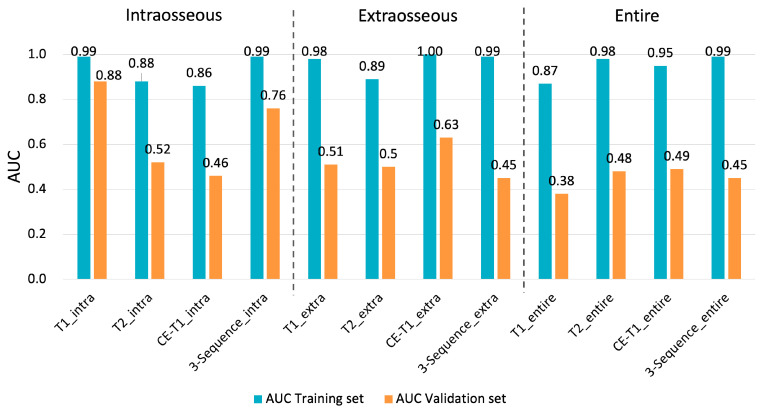
Diagnostic performance of the 12 texture models using the support vector machine (SVM) algorithm for the training and validation sets in predicting 3-year survival (non-survivors vs. survivors, 7 vs. 27). Model T1_intra is constructed using the texture features extracted from intraosseous lesions on the T1-weighted images. The other models are constructed similarly. Model T1_intra showed the highest diagnostic performance among the 12 models (AUCs of 0.99 and 0.88 in the training and validation sets).

Regarding the discrimination between the chondroblastic and non-chondroblastic groups, model T2_extra showed the highest diagnostic performance among the 12 models (AUCs of 0.94 and 0.89 in the training and validation sets) (Figure 4, Table S2). Regarding the discrimination between the non-survivor and survivor groups, model T1_intra showed the highest diagnostic performance among the 12 models (AUCs of 0.99 and 0.88 in the training and validation sets) (Figure 5, Table S3). We show the confusion matrix and key metrics for the main results, i.e., when applying model T2_extra to all the cases, in discriminating the chondroblastic pattern from non-chondroblastic patterns in Table S4. Similarly, we show the confusion matrix and key metrics for model T1_intra in predicting 3-year survival when applying it to all the cases in Table S5. The sensitivity, specificity, PPV, and NPV for discriminating the chondroblastic pattern from the non-chondroblastic pattern were 100%, 84.6%, 66.7%, and 100%, respectively. The sensitivity, specificity, PPV, and NPV for discriminating non-survivors from survivors were 85.7%, 92.6%, 75.0%, and 96.2%, respectively.

## 4. Discussion

In this study, 8 patients exhibited the chondroblastic pattern, while the remaining 26 had non-chondroblastic patterns. Of the total, 27 patients were 3-year survivors, and 7 were non-survivors. The model based on extraosseous lesions from the T2-weighted images demonstrated the highest diagnostic performance in distinguishing between chondroblastic and non-chondroblastic patterns, with AUCs of 0.94 and 0.89 in the training and validation sets, respectively. For differentiating 3-year non-survivors from survivors, the model using intraosseous lesions on the T1-weighted images showed the best performance, achieving AUCs of 0.99 and 0.88 in the training and validation sets. This model had a sensitivity of 85.7%, specificity of 92.6%, positive predictive value of 75.0%, and negative predictive value of 96.2%. The texture models from extraosseous lesions on the T2-weighted images were effective in distinguishing chondroblastic from non-chondroblastic patterns, while the texture models from intraosseous lesions on the T1-weighted images could differentiate between 3-year survivors and non-survivors. Our results initially revealed the usefulness of pretreatment MRI-based texture analysis based on intraosseous and extraosseous lesion compartments in predicting histological patterns and 3-year survival.

Model T2_extra could discriminate the chondroblastic pattern from non-chondroblastic patterns with the highest AUC of 0.89 among the 12 texture models in the validation set. Previous studies reported that the T2W sequence could demonstrate relatively dense hyaline cartilage components as hyper-intensity, osteoid and fibrotic components as hypo-intensity, and involved cortical bone as iso-intensity to hyper-intensity [52]. Geirnaerdt et al. reported that a moderate to high cellular cartilage component was observed in the peripheral areas compared to the central areas in chondroblastic pattern lesions, while non-chondroblastic patterns did not show these distinctive distributions [15]. These findings suggest that chondroblastic components tend to be present in extraosseous compartments. As previous studies have reported, the chondroblastic pattern was considered the worst type for response to chemotherapy or survival [2,53,54], although our study did not find a significant correlation between the chondroblastic pattern and 3-year survival (Table 1, *p* = 0.4997), possibly due to the small sample size, and further studies are warranted to determine the correlation between the chondroblastic pattern and survival in patients with OS. Models constructed on the T1W or CE-T1W sequences were not selected due to their lower AUCs in the validation sets. We speculated that the T1W or CE-T1W sequences might not be able to specifically reflect the distinctive distribution of these histological components. Regarding the lesion compartments, the “entire model” (T1_entire, T2_entire, and CE-T1_entire) was constructed using the PCs of a total of the texture features extracted from the entire OS lesion for each sequence (Figure 3). Despite the fact that the “entire model” has been used generally to perform texture analysis on OS lesions in previous studies [8,14,20,24,37], it was not selected in our study for the discrimination of histological patterns. When texture analysis is performed on the entire lesion, the texture features that reflect the distinctive distribution of the histological components of the peripheral areas might not be emphasised.

Model T1_intra could discriminate the 3-year non-survivors from survivors with the highest AUC of 0.88 among the 12 texture models. Furthermore, model T1_intra showed satisfactory sensitivity, specificity, PPV, and NPV of 85.7%, 92.6%, 75.0%, and 96.2%, respectively. In cases judged as non-survivors by model T1_intra, it could be considered an indication for receiving more aggressive preoperative chemotherapy or making decisions about the appropriate time for surgical resection [55]. Model T1_intra was constructed using the PCs explaining over 80% of the texture features from intraosseous lesions on the T1W images. OS originates in the intramedullary cavity and is present as an intraosseous lesion [56]. Intraosseous lesions show heterogeneous hypo-intensity to iso-intensity on T1W images with hyperintense haemorrhagic areas or hypointense ossified areas [9,16,57]. These imaging features on T1W images that appear with the growth of intramedullary OS lesions may reflect survival-related tumour aggressiveness [58,59,60,61]. Thus, model T1_intra could predict 3-year survival in patients with OS by quantifying these intraosseous imaging features. In our study, the “entire model” was not selected to predict 3-year survival due to its lower AUC in the validation set. We speculated that 3-year survival might depend on intramedullary OS origination, while extraosseous soft tissue lesions might have a different clinical significance from intraosseous lesions. Regarding the CE-T1W sequences, heterogeneous imaging features (e.g., enhancement in viable tumours, septal/nodular enhancement in chondroblastic components, and non-enhancement in ossified areas) present in intraosseous and extraosseous lesions [15]; however, the T2W and T1W sequences were sufficient to evaluate histological patterns and 3-year survival in our study. Although the usefulness of diffusion-weighted imaging (DWI) in the evaluation of soft tissue tumours has been reported, the European Society of Musculoskeletal Radiology Guidelines 2023 also shows that there is no complete agreement, with a level of agreement of 67% [62]. Therefore, DWI was not taken in the cases of this study, so it was not included in the analysis.

In the 3-year non-survivor group (*n* =7), 7 (100%) patients had lung metastases, while in the survivor group (*n* = 27), 14 (52%) patients had lung metastasis, and a significant difference was found in lung metastasis between the two groups (*p* = 0.0051). Lung metastasis was found to be the main factor affecting 3-year survival, and this result was consistent with previous studies [59,63].

Differences in contrast due to different magnetic field strengths (1.5 Tesla and 3 Tesla) can affect the T1 and T2 relaxation parameters, which can lead to bias in the results of texture analysis. In addition, discrepancies in resolution between different systems can also be a concern when considering the importance of consistent protocols in texture analysis. In this study, we did not adopt a harmonisation method aimed at reducing bias due to equipment and resolution, but in the future, we believe that it will be necessary to implement a method to improve the consistency of analysis by introducing a harmonisation method, such as image standardisation technology or deep learning-based correction, to adjust for differences between devices.

Feature reduction is necessary for reducing the dimensionality of datasets in texture analysis. In this study, we performed texture feature reduction using PCA. PCA is a method of dimensionality reduction based on the internal variability of the dataset without knowing the outcomes, and it enables us to create robust models with a small sample size. Selecting the appropriate areas or performing segmentation in intraosseous and extraosseous lesions might be time-consuming, but we revealed the distinctive usefulness of the texture analysis of different lesion compartments on the T1W and T2W images relating to the histological patterns and 3-year survival. Although we could not validate the two models in a separate dataset, models T2_extra and T1_intra might help facilitate treatment strategy planning in clinical practice. PCA is widely used as a method for efficiently compressing information between correlated features, so we decided that it would be suitable for the exploratory analysis in this study. However, other feature selection and reduction methods (e.g., Lasso regression, sequential feature selection, or selection methods using information gain) have also been used in other studies, and we believe that a comparison of these methods would also be beneficial. However, in this study, we prioritised verifying whether the region for extracting texture features was from extraosseous or intraosseous, so we did not apply other dimensionality reduction methods. We consider that research is needed to introduce these alternative methods and evaluate whether the performance and interpretability of the model can be improved.

This study was conducted to demonstrate the usefulness of MRI texture analysis, and we focused on the contribution of texture to the identification of tissue type and survival prediction. Therefore, other radiomics features such as shape and primary statistics (e.g., tumour length) were treated as outside the scope of the study. However, we believe that including these features in future studies may further improve the comprehensiveness and accuracy of the model. In addition, the 86 texture features extracted were selected in accordance with the Image Biomarker Standardisation Initiative standards, ensuring the standardisation and reproducibility of the analysis. In this study, we did not apply any filters to the preprocessing, and we extracted the features directly from the original images, but in the future, we think it is possible to further improve the accuracy of feature extraction by considering the application of filtering methods.

This study had several limitations. First, the sample size was relatively small due to the epidemiological nature of OS. Subgroup analysis (osteoblastic, chondroblastic, and fibrosblastic) was not performed. We were unable to validate the model performance in a separate validation set; instead, we performed a k-fold cross-validation. A limitation of this study is the inability to secure a separate test dataset due to constraints in the number of available cases. Second, we did not consider the prognostic effect of preoperative chemotherapy on survival. However, we proposed intraosseous or extraosseous MRI-based texture analysis to discriminate between different histological patterns and 3-year survival in patients with OS. Further studies are needed to evaluate the response rate to chemotherapy in patients or their influence on survival [64,65]. Third, one of the limitations of this study is the discrepancy between the imaging protocols before and after contrast. In this study, we used non-fat-suppressed T1W images and fat-suppressed T1W images after contrast for analysis. It will also be necessary to evaluate fat-suppressed T1W images before and after contrast in the future. Fourth, we did not perform a radiological–pathological comparison. In the future, a point-to-point correlation analysis between the texture features of model T2_extra and model T1_intra and histological components will be needed. Finally, manual segmentation was performed on the axial plane. One previous study showed a high similarity between the manual and semiautomatic segmentation of bone sarcomas on the axial plane. To facilitate MRI-based texture analysis to discriminate between different histological patterns and 3-year survival in patients with OS, the automatic segmentation of intraosseous and extraosseous OS lesions is desired.

## 5. Conclusions

In conclusion, the texture model derived from extraosseous lesions on the T2W images demonstrated utility in differentiating chondroblastic from non-chondroblastic patterns, whereas the texture model based on intraosseous lesions on the T1W images exhibited robust performance in distinguishing between 3-year survivors and non-survivors. Although this study is exploratory with a limited case sample, expanding the cohort in future research may facilitate the histological classification and prognostic assessment of osteosarcoma through MRI texture analysis. Such advancements hold the potential to contribute to enhanced survival outcomes in patients with osteosarcoma.

## Figures and Tables

**Table 1:** Comparison of the background characteristics of the patients between the non-survivor and survivor groups.

Groups (*N* = 34)		3-Year Survival		*p*-Value
		Non-Survivor (*n* = 7)	Survivor (*n* = 27)	
Sex (M/F)		0/7	10/17	0.0553
Age (Years)		40 (17–95)	27 (5−72)	0.3374
Tumour location	Femur	5 (71%)	21 (78%)	0.1676
	Tibia	0 (0%)	4 (15%)	
	Humerus	2 (29%)	2 (7%)	
Tumour length	<80 mm	1	6	0.6324
	>80 mm	6	21	
Lung metastasis (with/without)		7/0	14/13	0.0051 *
Time of detection of lung metastasis	First indication	0	2	0.2231
	During follow-up (CT/biopsy)	4/3	5/7	
Surgical resection	Tumour resection	3 (43%)	18 (67%)	0.2540
	Limb amputation	4 (57%)	9 (33%)	
Histological patterns (*n* (%))	Chondroblastic (*n* = 8)	1 (14%)	7 (26%)	0.4997
	Non-chondroblastic (*n* = 26)	6 (86%)	20 (74%)	
Chemotherapy	NECO-95J	0	1	0.5565
	JCGO0905	5	26	
AJCC staging at presentation	IIa (%)	1 (14%)	5 (19%)	0.7141
	IIb (%)	6 (86%)	20 (74%)	
	IVa (%)	0 (0%)	2 (7%)	
The time interval between pretreatment MRI and surgical resection (days)		204 ± 242	101 ± 77	0.2589

Results are expressed as mean ± standard deviation. *p*-value by Fisher’s exact test or the Wilcoxon signed-rank test. NECO-95J, neoadjuvant chemotherapy for osteosarcoma in Japan; JCGO0905, Japan Clinical Oncology Group; AJCC, American Joint Committee on Cancer staging. *Statistical significance is indicated (*p* < 0.05).

**Table 2:** Results of 12 support vector machine (SVM) models and parameters of selected principal components (PCs) by principal component analysis (PCA).

Lesion Compartment	SVM Models	Selected PCs	Eigenvalue	Contribution Ratio (%)	Cumulative Contribution Ratio (%)
Intraosseous lesions	T1_intra	PC1, PC2, PC3, PC4	14.04, 6.93, 4.67, 3.25	40.11, 19.81, 13.35, 9.28	82.55
	T2_intra	PC1, PC2, PC3	20.96, 5.31, 2.61	59.89, 15.16, 7.46	82.51
	CE-T1_intra	PC1, PC2, PC3, PC4	10.88, 7.94, 6.81, 3.34	31.09, 22.69, 19.46, 9.53	82.74
	3-Sequence_intra	PC1, PC2, PC3, PC4, PC5, PC6	30.12, 16.12, 13.02, 9.97, 6.61, 6.54	28.69, 15.35, 12.40, 9.50, 6.29, 6.23	78.46
Extraosseous lesions	T1_extra	PC1, PC2, PC3, PC4	13.04, 7.13, 4.49, 3.46	37.27, 20.37, 12.82, 9.87	80.33
	T2_extra	PC1, PC2, PC3, PC4	12.60, 6.59, 5.78, 3.50	35.99, 18.82, 16.52, 10.00	81.33
	CE-T1_extra	PC1, PC2, PC3, PC4, PC5	22.04, 12.71, 9.61, 5.90, 3.83	31.49, 18.16, 13.72, 8.42, 5.48	77.27
	3-Sequence_extra	PC1, PC2, PC3, PC4, PC5, PC6	31.81, 17.22, 12.39, 8.68, 7.55, 4.74	30.30, 16.40, 11.80, 8.27, 7.19, 4.52	78.47
Entire lesions	T1_entire	PC1, PC2, PC3, PC4	15.50, 7.17, 6.18, 4.8	36.04, 16.68, 14.38, 11.24	78.34
	T2_entire	PC1, PC2, PC3, PC4	17.27, 7.65, 6.55, 3.82	39.25, 17.39, 14.89, 8.68	80.21
	CE-T1_entire	PC1, PC2, PC3, PC4	16.65, 8.91, 5.15, 4.10	37.84, 20.24, 11.69, 9.32	79.30
	3-Sequence_entire	PC1, PC2, PC3, PC4, PC5, PC6, PC7	32.36, 20.71, 15.74, 9.93, 7.31, 6.94, 11.13	24.70, 15.81, 12.02, 8.50, 7.58, 5.58, 5.30	79.48

## Data Availability

The data that support the findings of this study are available upon request from the corresponding author.

## Supplementary Materials

The following supporting information can be downloaded at https://www.mdpi.com/article/10.3390/diagnostics14222562/s1, Table S1: Eighty-six texture features extracted from each tumour segmentation; Table S2: Diagnostic performance of the texture models using the support vector machine (SVM) algorithm for the training and validation sets in differentiating the chondroblastic pattern from non-chondroblastic patterns (8 vs. 26); Table S3: Diagnostic performance of the texture models using the support vector machine (SVM) algorithm for the training and validation sets to predict 3-year survival (survivors vs. non-survivors, 27 vs. 7); Table S4: The confusion matrix and diagnostic performance of Model T2_extra for all the cases in discriminating the chondroblastic pattern from the non-chondroblastic pattern; Table S5: The confusion matrix and diagnostic performance of Model T1_intra for all the cases in predicting 3-year survival.
