# Supplementary material for: Magnetic Resonance Imaging Texture Analysis Based on Intraosseous and Extraosseous Lesions to Predict Prognosis in Patients with Osteosarcoma
Source: Diagnostics (Basel). 2024 Nov 15;14(22):2562. doi: 10.3390/diagnostics14222562 (PMC11593140; doi:10.3390/diagnostics14222562)
Supplement: Supplementary file 1 [file diagnostics-14-02562-s001.zip › diagnostics-3267021-supplementary.pdf]

**Table S1.** Eighty-six texture features extracted from each tumour segmentation

| Metrices | Features |
|---|---|
| Conventional features | 1. Min |
| | 2. Mean |
| | 3. Std |
| | 4. Max |
| | 5. Q1 |
| | 6. Q2 |
| | 7. Q3 |
| | 8. Skewness |
| | 9. Kurtosis |
| | 10. Excess Kurtosis |
| | 11. Peak Sphere 0.5 mL: discretised volume sought |
| | 12. Peak Sphere 0.5 mL (value only for PET or MRI) |
| | 13. Peak Sphere 1 mL: discretised volume sought |
| | 14. Peak Sphere 1 mL (value only for PET or MRI) |
| | 15. Calcium Agatston Score [only for CT] |
| | 16. TLG (mL) [only for PET or MRI] |
| | 17. RIM_min |
| | 18. RIM_mean |
| | 19. RIM_stdev |
| | 20. RIM_max |
| | 21. RIM_Volume (mL) |
| | 22. RIM_Volume (vx) |
| | 23. RIM_sum |
| Discretised features | 1. Min |
| | 2. Mean |
| | 3. Std |
| | 4. Max |
| | 5. Q1 |
| | 6. Q2 |
| | 7. Q3 |
| | 8. Skewness |
| | 9. Kurtosis |
| | 10. Excess Kurtosis |
| | 11. Peak Sphere 0.5 mL: discretised volume sought |
| | 12. Peak Sphere 0.5 mL (value only for PET or MRI) |
| | 13. Peak Sphere 1 mL: discretised volume sought |
| | 14. Peak Sphere 1 mL (value only for PET or MRI) |
| | 15. TLG (mL) [only for PET or MRI] |
| | 16. RIM_min |
| | 17. RIM_mean |
| | 18. RIM_stdev |
| | 19. RIM_max |
| | 20. RIM_sum |
| | 21. HISTO_Skewness |
| | 22. HISTO_Kurtosis |
| | 23. HISTO_Excess Kurtosis |
| | 24. HISTO_Entropy_log10 |
| | 25. HISTO_Entropy_log2 |
| | 26. HISTO_Energy [=Uniformity] |
| Shape features | 1. Volume (mL) |
| | 2. Volume (vx) |
| | 3. Sphericity [only for 3DROI]) |
| | 4. Surface(mm2) [only for 3DROI] |

| | | |
|---|---|---|
| | 5. | Compacity [only for 3DROI] |
| Grey-Level Co-occurrence Matrix (GLCM) | 1. | Homogeneity [=Inverse Difference] |
| | 2. | Energy [=Angular Second Moment] |
| | 3. | Contrast [=Variance] |
| | 4. | Correlation |
| | 5. | Entropy_log10 |
| | 6. | Entropy_log2 [= Joint Entropy] |
| | 7. | Dissimilarity |
| Grey-Level Run Length Matrix (GLRLM) | 1. | Short Run Emphasis (SRE) |
| | 2. | Long Run Emphasis (LRE) |
| | 3. | Low Grey Run Emphasis (LGRE) |
| | 4. | High Grey Run Emphasis (HGRE) |
| | 5. | Short Run Low Grey Emphasis (SRLGE) |
| | 6. | Short Run High Grey Emphasis (SRHGE) |
| | 7. | Long Run Low Grey Emphasis (LRLGE) |
| | 8. | Long Run High Grey Emphasis (LRHGE) |
| | 9. | Grey-Level Non-Uniformity (GLNU) |
| | 10. | Run Length Non-Uniformity (RLNU) |
| | 11. | Run Percentage (RP) |
| Grey-Level Zone Length Matrix (GLZLM) | 1. | Short-Zone Emphasis (SZE) |
| | 2. | Long-Zone Emphasis (LZE) |
| | 3. | Low Grey-Level Zone Emphasis (LGZE) |
| | 4. | High Grey-Level Zone Emphasis (HGZE) |
| | 5. | Short-Zone Low Grey-Level Emphasis (SZLGE) |
| | 6. | Short-Zone High Grey-Level Emphasis (SZHGE) |
| | 7. | Long-Zone Low Grey-Level Emphasis (LZLGE) |
| | 8. | Long-Zone High Grey-Level Emphasis (LZHGE) |
| | 9. | Grey-Level Non-Uniformity (GLNU) |
| | 10. | Zone Length Non-Uniformity (ZLNU) |
| | 11. | Zone Percentage (ZP) |
| Neighbouring Grey-Level Difference Matrix (NGLDM) | 1. | Coarseness |
| | 2. | Contrast |
| | 3. | Busyness |

**Table S2.** Diagnostic performance of texture models using support vector machine (SVM) algorithm for training and validation sets in differentiating the chondroblastic pattern from non-chondroblastic patterns (8 vs. 26)

| Lesion compartment | SVM models | Principal component | AUC of training set | AUC of validation set |
|---|---|---|---|---|
| Intraosseous lesions | T1_intra | PC1-4 | 0.92 | 0.17 |
| | T2_intra | PC1-3 | 0.93 | 0.76 |
| | CE-T1_intra | PC1-4 | 0.89 | 0.48 |
| | 3-Sequence_intra | PC1-6 | 1.00 | 0.47 |
| Extraosseous lesions | T1_extra | PC1-4 | 0.98 | 0.72 |
| | T2_extra | PC1-3 | 0.94 | 0.89 |
| | CE-T1_extra | PC1-5 | 0.99 | 0.67 |

| | | | | |
|---|---|---|---|---|
| | 3-Sequence_extra | PC1-6 | 0.99 | 0.70 |
| Entire lesions | T1_entire | PC1-4 | 0.95 | 0.58 |
| | T2_entire | PC1-3 | 0.97 | 0.80 |
| | CE-T1_entire | PC1-4 | 0.98 | 0.57 |
| | 3-Sequence_entire | PC1-7 | 0.99 | 0.75 |

**Table S3.** Diagnostic performance of texture models using support vector machine (SVM) algorithm for training and validation sets to predict 3-year survival (survivors vs. non-survivors, 27 vs. 7)

| Lesion compartment | SVM models | Principal component | AUC of training set | AUC of validation set |
|---|---|---|---|---|
| Intraosseous lesions | T1_intra | PC1-4 | 0.99 | 0.88 |
| | T2_intra | PC1-3 | 0.88 | 0.52 |
| | CE-T1_intra | PC1-4 | 0.86 | 0.46 |
| | 3-Sequence_intra | PC1-6 | 0.99 | 0.76 |
| Extraosseous lesions | T1_extra | PC1-4 | 0.98 | 0.51 |
| | T2_extra | PC1-3 | 0.89 | 0.50 |
| | CE-T1_extra | PC1-5 | 1.00 | 0.63 |
| | 3-Sequence_extra | PC1-6 | 0.99 | 0.45 |
| Entire lesions | T1_entire | PC1-4 | 0.87 | 0.38 |
| | T2_entire | PC1-3 | 0.98 | 0.48 |
| | CE-T1_entire | PC1-4 | 0.95 | 0.49 |
| | 3-Sequence_entire | PC1-7 | 0.99 | 0.45 |

**Table S4.** The confusion matrix and diagnostic performance of Model T2_extra for all cases in discriminating the chondroblastic pattern from non-chondroblastic pattern

| Model T2_extra | Chondroblastic pattern (n =8) | Non-chondroblastic pattern (n =26) |
|---|---|---|
| Model Prediction Positive | 8 | 4 |
| Model Prediction Negative | 0 | 22 |

Sensitivity: 100%; Specificity: 84.6%; Positive predictive value: 66.7%; Negative predictive value: 100%

**Table S5.** The confusion matrix and diagnostic performance of Model T1_intra for all cases in predicting 3-year survival

| Model T1_intra | Non-Survivor (n = 7) | Survivor (n = 27) |
|---|---|---|
| Model Prediction Positive | 6 | 2 |
| Model Prediction Negative | 1 | 25 |

Sensitivity: 85.7%; Specificity: 92.5%; Positive predictive value: 75.0%; Negative predictive value: 96.2%
